# Dysglycemia in the critically ill patient: current evidence and future perspectives

**DOI:** 10.5935/0103-507X.20170054

**Published:** 2017

**Authors:** Ignacio Aramendi, Gastón Burghi, William Manzanares

**Affiliations:** 1 Centro Nacional de Quemados, Hospital de Clínicas Dr. Manuel Quintela, Facultad de Medicina, Universidad de la República - Montevideo, Uruguay.; 2 Cátedra de Medicina Intensiva, Hospital de Clínicas Dr. Manuel Quintela, Facultad de Medicina, Universidad de la República - Montevideo, Uruguay.

**Keywords:** Blood glucose, Hyperglycemia, Hypoglycemia, Hemoglobin A, glycosylated, Mortality, Severity of illness index, Intensive care units

## Abstract

Dysglycemia in critically ill patients (hyperglycemia, hypoglycemia, glycemic variability and time in range) is a biomarker of disease severity and is associated with higher mortality. However, this impact appears to be weakened in patients with previous diabetes mellitus, particularly in those with poor premorbid glycemic control; this phenomenon has been called "diabetes paradox". This phenomenon determines that glycated hemoglobin (HbA1c) values should be considered in choosing glycemic control protocols on admission to an intensive care unit and that patients' target blood glucose ranges should be adjusted according to their HbA1c values. Therefore, HbA1c emerges as a simple tool that allows information that has therapeutic utility and prognostic value to be obtained in the intensive care unit.

## Abstract (es)

La disglucemia en el paciente crítico (hiperglucemia, hipoglucemia, variabilidad de la glucemia y el tiempo en rango) es un marcador de severidad de la enfermedad crítica asociada a mayor mortalidad. Sin embargo, dicho impacto parece atenuarse en los pacientes con diabetes mellitus, en particular en aquellos con mal control glucémico premórbido lo cual ha sido denominado "paradoja de la diabetes". Este fenómeno determina que en los nuevos protocolos de control de la glucemia deban ser contemplados los valores de hemoglobina glucosilada (HbA1c) al ingreso a unidad de cuidados intensivos, siendo necesarios nuevos rangos de glucemia objetivos según los valores de la HbA1c. En tal sentido, la HbA1c surge como una herramienta sencilla que permite obtener información de utilidad terapéutica y valor pronóstico en la unidad de cuidados intensivos.

## INTRODUCTION

Intensive monitoring of plasma glucose levels and insulin treatment in the critical patient have been a standard of care in the intensive care unit (ICU) and an area of ongoing research during the last 15 years, following the publication of the pioneering study of van den Berghe et al. in 2001.^(1)^ This study, which involved 1,543 critical surgical patients, produced results that modified the way in which alterations in blood glucose levels are addressed in critical care. However, subsequent multicentric randomized controlled trials (RCTs) were not able to replicate the results of van den Berghe's initial study. In contrast, these RCTs showed unacceptable levels of hypoglycemia in some patients,^(2,3)^ and some of them, such as the Normoglycemia in Intensive Care Evaluation and Survival Using Glucose Algorithm Regulation (NICE-SUGAR), showed a significant increase in mortality in patients who received intensive treatment with insulin.^(4)^ On the other hand, in the last few years, the concept of dysglycemia in ICU has emerged; in a sense, this concept has made our understanding of glycemic alterations in critically ill patients more complex, although the concept has been used to explain the differences in the results obtained in RCTs. The objective of the present review is to describe the current state of knowledge concerning dysglycemia in the critically ill, to analyze its various components and domains, and to establish future strategies for the study and treatment of alterations of glycemia in ICU patients, in whom the determination of glycosylated hemoglobin would seem to be of paramount importance.

### Dysglycemia in the critical patient: definition and concept

So-called dysglycemia in the critically ill patient is a concept that encompasses four variables or domains of glycemic control: stress hyperglycemia itself, glycemic variability (GV), hypoglycemia, and another more recently recognized variable of prognostic importance, time in the target range (TITR).^(5)^ We will discuss the different domains of dysglycemia, detailing the therapeutic approach to and the prognostic impact of each.

### Stress hyperglycemia

Stress hyperglycemia, which is defined as a fasting blood glucose level greater than 126mg/dL measured on two successive occasions or a record higher than 200mg/dL at any point in its evolution, is a frequent disorder in hospitalized patients in the absence of previous diabetes mellitus (DM).^(6)^ Its etiological basis includes a series of events ranging from endogenous responses to stress to the consequences of therapeutic interventions such as catecholamine infusion and the use of parenteral dextrose and glucocorticoids. This form of hyperglycemia is also the consequence of a series of immunoinflammatory and hormonal alterations that characterize critical illness and systemic inflammation, such as the presence of proinflammatory cytokines and increased levels of hormones that are counterregulatory to insulin (glucagon, cortisol, catecholamines and growth hormone). These changes lead to increased hepatic glucose generation (neoglucogenesis and glycogenolysis) as well as to peripheral resistance to the action of insulin.^(7)^

### Hypoglycemia

Hypoglycemia, both spontaneous and secondary to treatment with insulin, is an extremely frequent finding in the ICU and has been linked to increased mortality of critically ill patients.^(8)^ The risk factors for its development include sepsis, DM, severe critical illness, renal injury or hepatic dysfunction, requirement for vasoactive drugs, and suspension of nutritional therapy during insulin infusion.^(8)^ However, as demonstrated by Yamada et al.,^(9)^ the risk factor most commonly associated with hypoglycemia is intensive insulin therapy. In that study, intensive insulin therapy was associated with a five-fold elevation in the risk of severe hypoglycemia (glycemia < 40mg/dL) (p < 0.001).^(9)^

On the other hand, the development of hypoglycemia has been the most frequent complication in the glycemic control RCTs reported to date.^(1-3)^ The authors of the NICE-SUGAR study^(4)^ analyzed the correlation between the presence of moderate (40 - 70mg/dL) and severe (less than 40mg/dL) hypoglycemia and mortality. Of the 6,026 patients analyzed, 45% experienced hypoglycemia (82.4% in the intensive group with insulin). The mortality rate in patients without hypoglycemia was 23.5%, whereas it was 28.5% and 35.4% in patients with moderate and severe hypoglycemia, respectively.^(10)^ Similar findings were observed in both of the RCTs mentioned earlier as well as in a number of observational studies.^(11)^ The presence of at least one episode of mild hypoglycemia (40 - 69mg/dL) was shown by Krinsley et al. in 2011^(12)^ to be associated with increased length of stay in the ICU. Further analysis of the same group of patients revealed that the occurrence of at least one episode of mild hypoglycemia (55 - 69mg/dL) was significantly associated with an increased risk of death (p < 0.0001).^(13)^

### Glycemic variability

The third domain of glycemic control in the ICU is GV. In critically ill patients, including those receiving continuous nutrition and insulin infusion, plasma glucose levels fluctuate very markedly. Thus, in presence of similar mean blood glucose values, glycemic control may differ significantly according to the existing GV.^(14)^ To date, the definition of GV and the best method of reporting it have not been agreed upon. Conceptually, Braithwaite^(15)^ defined GV as *"the tendency or propensity of a patient to develop repeated excursions of plasma glycemia over a relatively short period of time which exceeds the range expected for a normal physiological response"*.^(15)^

The expression "GV" is ambiguous and includes different methods of measuring variability; these include but are not limited to a) the magnitude of glycemic excursions over a given time interval in relation to the mean plasma glucose level and b) the frequency with which a critical value is exceeded in a certain period of time.^(16)^

The first report on GV dates back to 2006, when Egi et al., studying a retrospective cohort from four centers (n = 7,049), defined GV as the standard deviation of glycemia and indicated it as an independent biomarker of mortality.^(17)^ Later, in 2008, Krinsley et al.^(18)^ analyzed the phenomenon of GV in 3,252 critical patients, grouping them according to their mean glycemic values during their stays in the ICU; the patients in each group were further divided into quartiles based on the standard deviation of their plasma glucose values. Analysis of the data showed a progressive and significant increase in mortality as the standard deviation of the patients' plasma glucose values increased, and this association was more evident in patients with average glycemic values in the euglycemic range (70 - 99mg/dL).^(18)^ It is also interesting to note that a significant number of observational studies published since this report have confirmed the existence of an association between GV and mortality.^(19,20)^ Thus, it is important at this point to attempt to establish explanations for this association as well as to determine whether GV is in itself a biomarker of severity with a deleterious biological effect or whether it constitutes a biomarker of the quality of care received in the ICU.

One hypothesis regarding the association of GV with increased mortality is that abrupt changes in glycemia trigger oxidative stress in patients with previous DM. Such oxidative stress can result in endothelial dysfunction and vascular damage. On the other hand, it has been observed that GV increases the adhesion of monocytes to endothelial cells in animal models and that it increases apoptosis in human cell cultures.^(21)^

Few studies have shown GV as a biomarker of mortality that is independent of the severity of underlying disease and of the strategy used for glycemic control. On the other hand, the results of published studies, most of which have been observational and retrospective, show great heterogeneity; also, the diversity of ways in which GV is measured adds confusion and hinders the overall interpretation of the data. For the above, it will be necessary to elucidate in the near future whether GV is simply a biomarker of severity or whether, on the contrary, it is a cause of death in critical illness.

### Time in target range: the unifying domain

Time in target range has been introduced recently as the fourth or "unifying" domain of dysglycemia in ICU patients.^(5)^ Time in target range is defined as the accumulated time in the target band and expresses the percentage of time in which a patient's glycemic level remains within the target range. Conceptually, TITR may vary from patient to patient despite the presence of similar blood glucose levels. This variation could constitute a confounding factor that could explain the negative results of strict glycemic control in the large RCTs published to date.^(5)^ However, TITR has not been analyzed as a variable in the RCTs that have been published to date. In a retrospective analysis, Signal et al.^(22)^ evaluated the relationship between a TITR of 71 - 126mg/dL and hospital mortality. In the same study, the presence of a TITR greater than 70% was significantly associated with an increase in survival in critical illness.^(22)^ The same group performed a *post hoc* analysis of data from the GluControl study,^(23)^ a study that had to be finalized prematurely as a consequence of protocol violations and difficulty in reaching the target ranges for glycemic control. In the analysis, which included patients from both the intervention and the control groups, the presence of a TITR greater than 50% was associated with greater survival.^(23)^ In 2015, in a study of patients undergoing cardiac surgery, Omar et al.^(24)^ reported that the presence of a TITR greater than 80% was associated with a significant reduction in postoperative atrial fibrillation as well as with shorter mechanical ventilation time, shorter ICU stay and lower incidence of infection of the operative wound.^(24)^

Recently, Krinsley and Preiser^(25)^ analyzed the relationship between a TITR of 70-140 mg/dL and mortality. This analysis is of particular importance because theirs is so far the only study that has examined the TITR in relation to the presence or absence of previous DM. In this analysis, the mortality of non-diabetic patients doubled when TITR was less than 80% (15.7% mortality and 8.4% mortality in patients with TITR less than and greater than 80%, respectively; p = 0.0001).^(25)^ However, TITR was not associated with mortality in the group of diabetic patients. With respect to the interaction between TITR and the other three domains of glycemic control, the authors demonstrated a strong association between the domains of dysglycemia and mortality in patients without DM, including patients with high TITR. However, this association was not observed in patients with previous DM.^(25)^ Therefore, it can be stated that TITR, along with glycemic control and GV prevention strategies, should be a therapeutic objective in current glycemic control protocols.

### Importance of diabetes mellitus: *"The Diabetes Paradox"*

The early work of the Leuven group included the observation that intensive treatment with insulin had a greater benefit in patients without DM than in patients with known DM. Similar results have been reported in a number of significant RCTs (Table 1).^(26)^ These findings coincide with a growing body of evidence derived from observational studies that points to DM as a protective factor in the critical patient.^(31,32)^ In a systematic review of the literature and meta-analysis published in 2011, Siegelaar et al.^(33)^ evaluated the impact of DM (18.6% of patients studied) on hospital mortality and at 30 days in a population of 12,489,574 critical patients. However, this analysis did not show an association between DM and mortality except in the group of heart surgery patients.^(33)^ This finding contrasts with the fact that DM is associated with an increase in morbidity and mortality in outpatients.^(34)^ The apparently protective effect of DM in critical illness has been called the *"diabetes paradox"*.^(35)^

**Table 1:** Clinical trials on intensive *versus* conventional glycemic control and most outstanding outcomes. We highlight the analysis of the mortality discriminated according to the presence or absence of previous diabetes mellitus

Clinical trial	Study population	Patients with DM (%)	Target range (mg/dL) Intensive versus conventional group	Mean glycemia (mg/dL) Intensive versus conventional group	Mortality (%) Intensive/conventional group	Other results (Intensive versus conventional group)
van den Berghe et al.^(1)^	Surgical (n = 1548)	204 (13.2)	80 - 110 180 - 200	103 153	Overall (4.6/8.0) (p = 0.04) Without DM (4.7/8.4) With DM (4.0/5.8)	Lower incidence of bacteremia (4.2% *versus* 7.8%, p = 0.003); TRR (4.8% *versus* 8.2%, p = 0.007); PNP (28.7% *versus* 57.9%, p = 0.001) VM (d) (10 *versus* 12, p = 0.006)
van den Berghe et al.^(27)^	Medical (n = 1200)	203 (16.9)	80 - 110 180 - 200	111 153	Overall (24.2/26.8) (p = 0.31) Without DM (36.8/40.9) With DM (39.6/35.0)	Shorter MV time (HR 1.21, 95%CI 1.02 - 1.44, p = 0.03); ARF (5.9% *versus* 8.9%, p = 0.04); Staging in ICU (HR 1.15, 95% CI 1.01-1.32, p = 0.04)
NICE-SUGAR^(4)^	Medical-surgical (n = 6104)	1211 (20.1)	80 - 108 < 180	118 145	Overall (27.5/24.9) (p = 0.02) Without DM (26.5/24.2) With DM (31.7/27.7)	Severe hypoglycemia (6.8% *versus* 0.5%, p = 0.001)
Arabi et al.^(28)^	Medical-surgical (n = 523)	208 (39.8)	80 - 110 180 - 200	115 171	Overall (13.5/17.1) (p = 0.70) Without DM (13.8/14.2) With DM (12.9/20.3)	Hypoglycemia (28.6% *versus* 3.1%, p = 0.0001)
De la Rosa et al.^(29)^	Medical-surgical (n = 504)	61 (12.1)	80 - 110 180 - 200	117 149	Overall (36.6/32.4) With DM (37.5/31.0)	Severe hypoglycemia (8.3% *versus* 0.8%, p = 0.001)
Brunkhorst et al.^(3)^	Sepsis/septic shock (n = 488)	163 (30.4)	80 - 110 180 - 200	112 151	24.7 *versus* 26.0 (p = 0.74)	Severe hypoglycemia (17.0% *versus* 4.1%, p = 0.001)
Preiser et al.^(2)^	Medical-surgical (n = 1078)	203 (18.8)	80 - 110 140-180	118 145	17.2 *versus* 15.3 (p = 0.41)	Severe hypoglycemia (8.7% *versus* 2.7%, p = 0.0001)
Kalfon et al.^(30)^	Medical-surgical (n = 2684)	536 (20.2)	80 - 110 < 180	115 126	32.3 *versus* 34.1 (p = 0.32)	Severe hypoglycemia 13.2% *versus* 6.2%, p = 0.001)

DM - diabetes mellitus; HR - hazard ratio; CI - confidence interval; IRA - acute renal injury; TRR - renal replacement therapy; ICU - intensive care unit; VM - mechanical ventilation.

On the other hand, observational studies have demonstrated a relationship among the presence of DM, the three domains of glycemic control and mortality. In 2013, Krinsley et al.^(36)^ showed that the diagnosis of DM correlated with a decreased risk of death. However, hyperglycemia was significantly associated with an increase in mortality in non-diabetic patients but not in those with DM.^(36)^ Finally, the authors demonstrated that increased GV was significantly associated with an increase in mortality in non-diabetic patients but not in patients with DM.^(36)^

A retrospective analysis (n = 3,529) comparing patients who followed a strict control protocol (80 - 110mg/dL) with patients under moderate control (90 - 140mg/dL) showed that moderate control was associated with an increased risk of death in patients without DM (p < 0.05) and with a lower risk of death in diabetic patients (p = 0.01).^(37)^

The above findings suggest that the presence of DM can modulate the effects of glycemic control variables on critically ill patients. In this sense, we should ask ourselves two questions: a) What is the biological explanation that underlies these paradoxical findings in the presence of DM? and b) What is the possible impact of glycemic control prior to admission to the ICU?

Regarding the first point, there is no conclusive evidence to explain the diabetes paradox. However, various authors, including Klip et al.,^(38)^ have postulated that chronic hyperglycemia results in cellular conditioning that is protective against the damage caused by acute hyperglycemia during critical illness. The mechanism underlying such conditioning might that chronic exposure to hyperglycemia causes downregulation of the GLUT 1 and GLUT 3 transporters, which are stimulated during the acute phase of critical illness and whose activity contributes to the toxicity of cellular glucose overload.^(38)^ Additionally, factors not related to glycemic control, such as the existence of DM and the potential beneficial effects of insulin (anti-inflammatory and endothelial protective effects), may in part explain the so-called diabetes paradox. In this regard, clinical and animal studies indicate that DM is a protective factor against the development of acute respiratory distress syndrome (ARDS);^(39)^ this phenomenon is mediated by, among other factors, an increase in the expression of peroxisome proliferator-activated receptor gamma (PPAR-γ), which may be overexpressed in patients with DM.

However, there is strong evidence demonstrating the relationship between the control of premorbid glycemia and dysglycemia. In a retrospective analysis of 415 diabetic patients admitted to the ICU, the value of glycosylated hemoglobin (HbA1c) was determined during the three months prior to admission.^(40)^ The authors observed that in patients with poorer prior control (HbA1c > 6.8%), mortality was higher when weighted mean time of plasma glucose (GLUtw) (and therefore glycemic control) was better during the stay in the ICU. The multivariate analysis found a significant correlation between HbA1c and GLUtw, noting that the relationship between HbA1c and mortality was modified according to changes in GLUtw (p = 0.008).^(40)^ These results indicate that patients with poorer metabolic control prior to ICU admission had greater survival when ICU blood glucose values were higher and that their survival was lower when their ICU blood glucose values approached euglycemic levels. On the other hand, in diabetic patients with better metabolic control prior to ICU admission, survival was higher when blood glucose values were lower.^(40)^

More recently, in an interesting cohort study described in 2014, Plummer et al.^(41)^ prospectively analyzed 1000 critically ill patients. The authors analyzed the relationship between mortality and premorbid glycemic control through the determination of HbA1c at ICU admission and measurement of peak blood glucose within the first 48 hours of admission. The patients in the study were classified into four categories: known diabetic based on the patient's clinical history; DM not known but currently indicated by an HbA1c value greater than 6.5%; patients with stress hyperglycemia; and patients who were normoglycemic.^(41)^ In patients with stress hyperglycemia and in those with DM with very good metabolic control (HbA1c < 6%) and adequate prior metabolic control (HbA1c between 6 and 7%), the increase in peak blood glucose in the first 48 hours in ICU was significantly associated with an increase in mortality. However, in known or unknown diabetic patients with poor previous metabolic control (HbA1c > 7%), the increase in peak blood glucose in the first 48 hours of evolution was not associated with higher mortality.^(41)^ In a retrospective analysis of 1,569 diabetic patients recently reported by the same group,^(42)^ glycemic variability was significantly associated with increased mortality (p = 0.001); however, this association was not observed in diabetic patients with poor metabolic control (HbA1c > 8.5%).^(42)^ Another retrospective study showed that poor premorbid metabolic control was significantly associated with higher mortality in diabetic patients with severe hypoglycemia during ICU stay.^(43)^

These findings suggest that blood glucose levels that may be safe and desirable for some groups of patients may not be safe in diabetic patients with previous poor metabolic control or chronic hyperglycemia. Based on the above, glycemic control prior to ICU admission could be a key factor in the development of glycemic control protocols.

### Glycosylated hemoglobin: importance in the critically ill

HbA1c is a biomarker of long-term glycemic control in diabetic patients; it reflects glycemic control in the three months prior to its determination.^(44)^ The American Diabetes Association (ADA) introduced a cutoff value of 6.5% as a diagnostic criterion for diabetes,^(45)^ and diabetes management guidelines suggest a value lower than 7.0% as an indicator of adequate glycemic control.^(46)^

In the field of intensive care medicine, the determination of HbA1c on admission has shown prognostic value. In a heterogeneous population of critical patients with no previous history of DM, HbA1c values greater than 6.5 were associated with greater severity and mortality in the ICU.^(47)^

The diagnosis of DM in the ICU is usually performed based on the patient's clinical history, but a significant number of patients undoubtedly have undiagnosed DM at the time of ICU admission.^(47,48)^ In an interesting prospective observational study by Carpenter et al. that included 15,737 critical patients, it was observed through HbA1c determination that 9% of the patients analyzed had unknown DM at the time of admission. This subgroup of patients demonstrated not only a significant increase in dysglycemia but also higher mortality (13.8% *versus* 11.4%; p = 0.01) compared to patients without DM.^(48)^

The determination of HbA1c at admission allows patients with stress hyperglycemia to be discriminated from those with DM and hyperglycemia. A number of observational studies have determined the prognostic value of the so-called "glycemic gap" (GG) in heterogeneous populations of critically ill diabetic patients. GG is defined by the difference between glycemia at admission to the ICU and estimated mean glycemia determined from the HbA1c value (GG = 28.7 x HbA1c - 46.7).^(49)^ GG has emerged as a predictor of adverse outcomes in diabetic patients with community-acquired pneumonia,^(50)^ myocardial infarction,^(51)^ and hepatic abscesses.^(52)^ In addition, values of GG > 80mg/dL have been associated with increased hospital mortality in critically ill diabetic patients, and their incorporation into the APACHE II score has increased its performance as a predictor of mortality.^(53)^ A somewhat similar measure, the so-called stress hyperglycemia ratio (SHR), is defined as the ratio between glycemia at admission/mean glycemia from HbA1c. This index has been postulated to be a more precise biomarker of metabolic stress than absolute hyperglycemia.^(54)^

In relation to the above, the determination of HbA1c at ICU admission can be of inestimable value when designing glycemic control protocols, considering that it was recently demonstrated that HbA1c levels are not altered during the onset of critical illness.^(55)^

### Target ranges of glycemia in different critical patient groups

In 2014, the American College of Physicians recommended maintaining glycemia within the range of 140 - 200mg/dL regardless of the patient's previous history of DM.^(56)^ The Society of Critical Care Medicine (SCCM) has recommended that infusion of insulin should be initiated when glycemia values are greater than 150mg/dL and that absolute values above 180mg/dL should be avoided.^(57)^ More recently, the guidelines of the American Diabetes Association recommended a target range of 140 - 180mg/dL for most hospitalized patients, critical or not.^(58)^ However, as previously stated, in our opinion the target ranges should be considered based on the presence or absence of DM in the patient. In an elegant prospective study recently reported by Kar et al.,^(59)^ a "liberal" and a "standard" strategy (glycemic targets of 14mmol/L and 10mmol/L, respectively) were sequentially compared in patients with T2DM who had poor metabolic control (HbA1c > 7.0% at admission). The liberal strategy was associated with a non-significant decrease in the relative risk of presenting episodes of moderate to severe hypoglycemia (p = 0.09). However, the liberal strategy significantly reduced (p < 0.01) the measured GV according to the coefficient of variability.^(59)^ In another interesting study recently reported by Di Muzio et al.,^(60)^ the researchers performed a prospective analysis of 80 critical patients by applying a conventional strategy (glycemic target: 6-10 mmol/L) or a later liberal strategy (10 - 14mmol/L). Patients subjected to the liberal strategy had a lower relative number of episodes of hypoglycemia as determined by a decrease in blood glucose to below 30% of the premorbid average glycemic value estimated from HbA1c at ICU admission.^(60)^

In a recently published interventional study,^(61)^ the efficacy and safety of a single strategy ("before") with a target range of 90 - 120mg/dL were compared with those of a differential strategy ("after") based on the patients' history of DM and HbA1c; in the differential strategy, target ranges of 80 - 140mg/dL and 110 - 160mg/dL were used for patients whose HbA1c values were below and above 7%, respectively. Mortality was almost the same in the diabetic and non-diabetic patients; however, in the diabetic patient group with HbA1c greater than 7%, the liberal strategy (target range 110 - 160mg/dL) was associated with a non-significant decrease in mortality and a significant decrease in the O:E mortality ratio.^(61)^

The clinical trials described above, which present various methodological weaknesses (limited numbers of patients, absence of randomization, unicentric character) are nevertheless the first trials to have explored the differential management of hyperglycemia in critically ill diabetic patients.

To develop a practical approach, we believe that it is necessary to modify the correction thresholds and the therapeutic ranges used when treating critically ill diabetic patients. In this sense, Marik et al.^(62)^ have suggested adopting a therapeutic range of 140 to 200mg/dL for diabetic patients with HbA1c < 7% on admission and a therapeutic range of 160 to 220mg/dL in those with HbA1c > 7%; this appears to be appropriate based on the available information.^(62)^ However, new clinical trials will be needed to validate these observations and to define whether or not a liberal glycemic control strategy is associated with better outcomes in critically ill diabetic patients with acute hyperglycemia.

**Figure 1:**
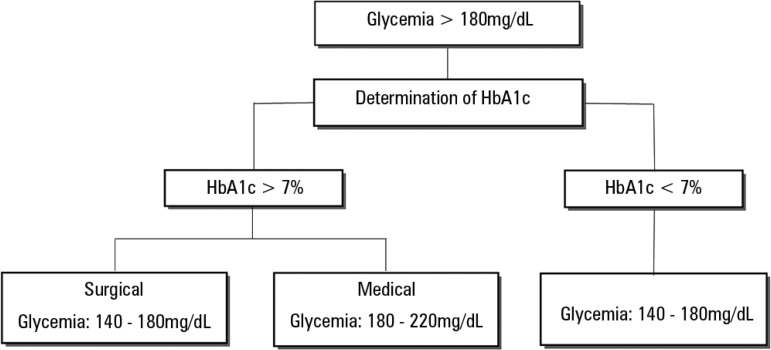
Algorithm of target glycemic ranges in critical patients with hyperglycemia according to glycosylated hemoglobin at admission to an intensive care unit. HbA1c - glycosylated hemoglobin.

Under the current state of knowledge, critically ill patients with hyperglycemia cannot be treated as a homogeneous group. For this reason, in our ICU we have implemented a new glycemic control protocol that requests HbA1c determination in all patients with glycemia > 180mg/dL (Figure 1). This allows a first diagnostic approach (stress hyperglycemia or previous DM) and the application of two protocols with different target ranges (180 - 220mg/dL in patients with HbA1c > 7% and 140 - 180mg/dL in patients with HbA1c < 7%).

## CONCLUSIONS

There is sufficient evidence to indicate that the four domains of dysglycemia in the critically ill patient are independent biomarkers of mortality. On the other hand, recent knowledge indicates that dysglycemia has a lower prognostic impact in patients with diabetes mellitus than in patients in whom diabetes mellitus is absent. Similarly, critically ill patients without diabetes mellitus or diabetic individuals in whom prior metabolic control has been adequate may benefit from tighter control with lower target glycemia levels. In that sense, the new protocols should consider the patient's premorbid glycemic control by determining his or her HbA1c. Finally, the so-called "time in range" has emerged as the unifying domain of glycemic control and has been shown to be both a prognostic biomarker and a quality-of-care biomarker in critically ill patients. In the near future, clinical research should be able to determine the true impact of diabetes mellitus on dysglycemia in the critically ill, as well as to consider new technologies to optimize blood glucose monitoring.
